# Fermentation of cigar tobacco leaves with citrus flavonoids: changes in chemical, microbiological, and sensory properties

**DOI:** 10.3389/fbioe.2024.1469532

**Published:** 2024-12-09

**Authors:** Wanrong Hu, Wen Cai, Yun Jia, Qianying Zhang, Zhengcheng Zhang, Yue Wang, Chongde Sun, Dongliang Li

**Affiliations:** ^1^ China Tobacco Technology Innovation Center for Cigar, China Tobacco Sichuan Industrial Co., Ltd., Chengdu, China; ^2^ Cigar Fermentation Technology Key Laboratory of Tobacco Industry, China Tobacco Sichuan Industrial Co., Ltd., Chengdu, China; ^3^ Industry Efficient Utilization to Domestic Cigar Tobacco Key Laboratory of Sichuan Province, Shifang, China; ^4^ Digital Intelligence Technology Research Center for Special Crops, Zhejiang University, Hangzhou, China

**Keywords:** cigar, fermentation, citrus extracts, chemical composition, community succession, sensory quality

## Abstract

**Introduction:**

Fermentation plays a significant role in improving the quality of cigar tobacco leaves. Particularly, fermentation with characteristic additives has been identified as an effective approach to enhance the fermentation process. The objective of this study was to develop new additives and investigate their influence on cigar tobacco leaves.

**Methods:**

The active ingredients extracted from three citrus species were obtained by ultrasonic-assisted extraction followed by solid phase purification. The effects of these ingredients as fermentation additives on the primary chemical components, microbial communities, and sensory quality of cigar tobacco leaves were subsequently analyzed.

**Results:**

Results showed that: (1) Flavonoids were the main components of the prepared citrus extracts. (2) The addition of citrus extracts resulted in a reduced content of amino acids, oxalic acid, and unsaturated fatty acids in cigar tobacco leaves, while increasing the content of citric acid. Besides, the total amount of aroma substances in cigar tobacco leaves increased by 38.15% with the addition of citrus extracts, with notable enhancements in the levels of *D*-limonene, β-dihydroionone, dihydroactiniolactone, and other representative aroma components. (3) The addition of citrus extracts promoted the succession of the microbial community in cigar tobacco leaves and promoted the enrichment of *Pseudomonas* and *Corynebacterium*. (4) The addition of citrus extracts effectively reduced the irritation and improved the aroma richness of cigar tobacco leaves.

**Discussion:**

In this study, the influence of citrus-derived active ingredients on cigar quality was systematically analyzed, providing a reference for the development of characteristic fermentation additives for cigars and the extension of cigar quality enhancement technologies.

## 1 Introduction

Fermentation is a crucial process in the treatment of cigar tobacco leaves (CTLs). After harvesting and air-curing, CTLs still exhibited several imperfections, such as obvious odor and irritation, insufficient maturity, and weak aroma (Wu et al., 2023). During fermentation, owing to the biochemical reactions involving microorganisms, enzymes, and chemical compounds in CTLs, improved maturity and decreased irritation were found (Zhang et al., 2023). Consequently, the appearance index, physical properties, chemical composition, and sensory quality of CTLs were enhanced. However, this process often relies on artificial intervention rather than occurring naturally.

At present, various methods have been developed to improve the efficiency of the fermentation process, such as adjusting the fermentation conditions and fermentation with exogenous additives. In the fermentation of tobacco leaves, common exogenous additives included a range of plant extracts, microbial agents, and enzyme preparations (Jia et al., 2023; Zheng et al., 2022b). Among them, plant extracts have garnered significant attention for its advantageous performances on storage and quality improvement in tobacco. Theoretically, plant extracts not only leverage their own flavor components but also affect the activity of microorganisms, thereby altering the quality of cigars (Hu et al., 2022). Previous studies have demonstrated that the direct addition of extracts from chrysanthemum, clove, coffee, cocoa, or fried rice can impart advanced properties to CTLs, such as enhanced flavor and sweetness (Hu et al., 2023b; Ren et al., 2024). However, these additives are typically crude extracts of plants or mixtures of chemical reagents. There is limited literature on the extraction of specific active ingredients from plants and their application in the fermentation of CTLs. Consequently, the development of other environmentally friendly and function-oriented additives appears to be a promising avenue for research.

Citrus refers to a group of plants classified under the genus *Citrus* within the family Rutaceae, which is characterized by the highest yield and consumption globally. Citrus fruits are abundant in carotenoids, carotene, essential oils, pectin, flavonoids, limonin, and various other bioactive compounds, demonstrating significant antioxidant and anti-inflammatory properties (Zhu et al., 2023). Flavonoids, which are secondary metabolites of polyphenols found in citrus, play a crucial role in the regulation of fruit color and flavor development (Wang et al., 2020). Similarly, tobacco also contains a variety of flavonoid compounds, including chlorogenic acid, coumarins, and flavonoids, which are important for the smoke flavor and visual appeal of tobacco leaves (Gao et al., 2019). Consequently, it can be hypothesized that the incorporation of flavonoids derived from citrus may positively influence the quality of CTLs. However, research focused on improving the quality of CTLs through the addition of citrus extracts remains limited. Furthermore, there is a paucity of knowledge regarding the influence of citrus extracts on the chemical composition, microbial community, and sensory quality of CTLs.

In this study, three varieties of citrus were selected as raw plant materials. Ultrasonic-assisted extraction, in conjunction with solid-phase extraction technology, was employed to obtain the citrus extracts. The chemical, microbiological, and sensory characteristics of CTLs fermented with different citrus extracts were investigated. The objective of this study was to provide a reference for the development of exogenous additives for cigars and to enhance technologies aimed at improving cigar quality.

## 2 Materials and methods

### 2.1 Extraction experiment

Three varieties of citrus fruits were procured from a local supermarket in China, as detailed in Table 1. The citrus fruits underwent lyophilization utilizing a freeze-dryer (SCIENTZ-10N, China) for 48 h, after which they were ground to a particle size of less than 250 μm. Ultrasound-assisted extraction of the citrus powder was conducted using anhydrous ethanol as the extraction solvent, with a solid-liquid ratio established at 1:10. The extraction process was carried out at a frequency of 53 kHz for a period of 1 hour. Following an 8-h period of natural sedimentation, the clarified filtrate was obtained by filtration through four layers of gauze and three layers of filter paper. The obtained filtrate was concentrated using a rotary evaporator (Buchi R-300, Switzerland) under reduced pressure until an ethanol-free phase was achieved (35°C, 80 rpm). Subsequently, double-distilled water was introduced to the residual liquid for redissolution. The aqueous phase was then collected for further purification.

**TABLE 1:** Nomenclature and raw material information of plant extracts.

Sample	Plant raw material	Part of plant	Region of plant
S1	*Citrus japonica Thunb*	ripe fruit	Guangxi, China
S2	*Citrus maxima* cv. *Tomentosa*	The outer rind of an immature fruit	Guangdong, China
S3	*Citrus Yuzu* L	ripe fruit	Zhejiang, China

The purification process was conducted using Solid Phase Extraction (SPE) with a Sep-pak^®^ C18 column. In a typical procedure, two bed volumes (BV) of methanol and 2 BV of water were used to activate the SPE columns. Subsequently, 0.75 BV of the sample supernatant was added, followed by the application of 20 BV of water to eliminate impurities such as sugars and acids. The SPE column was then eluted with 1 BV of methanol. The methanol in the eluate was subsequently volatilized using a rotary evaporator. The remaining unvolatilized residue constituted the citrus extract, which was utilized in subsequent fermentation experiments and stored at −20°C. Consequently, three citrus extracts were obtained, designated as S1, S2, and S3 (see Table 1).

### 2.2 Characterization of extracts

The detection of chemical components in citrus extracts was conducted using ultra-performance liquid chromatography coupled with electrospray ionization tandem mass spectrometry (UPLC-ESI-MS/MS), utilizing a UPLC system in conjunction with a Triple TOF 5600 mass spectrometry system.

Separation was performed on a UPLC system (1,290 Infinity, Agilent, United States) equipped with a Waters BEH C18 column (Acquity UPLC, 2.1 × 150 mm) maintained at 25°C. The injection volume was set at 2 μL, and the scanning wavelength was 280 nm. Optimal chromatograms were achieved employing a mobile phase of water (eluent A) and acetonitrile (eluent B) at a flow rate of 0.2 mL min^-1^, following a specific gradient elution program. Initially, the column was equilibrated with 5% eluent B for 2 min. The proportion of eluent B was linearly increased from 5% to 28% in 14 min, followed by a further linear increase to 60% in 4 min. In the next 6 minutes, the proportion of B increased to 100% and maintained this level for 2 min. Prior to the next injection, the proportion of B was reduced linearly to 5% in 2 min. The overall time between two consecutive injections was 30 min.

The MS was operated both in ESI+ and ESI−. The primary operational parameters of MS were set as follows: curtain gas (CUR) at 35 psi; ion spray voltage floating at 5.5 kV (−4.5 kV in ESI−); scanning range, 100–1,500 m/z; atomizing gas (GS1), 55 psi; atomizing gas (GS2), 55 psi; ion spray temperature (TEM), 600°C (ESI+) and 550°C (ESI−); primary scanning, declustering potential (DP) of 100 V and collision energy (CE) of 10 V; secondary scanning, TOF MS modes with information-dependent acquisition (IDA) and collision-induced dissociation (CID) energy of 40 ± 20 eV.

### 2.3 Fermentation experiment

A representative variety of CTL, specifically Dexue No. 7, was selected and sorted prior to the fermentation experiments to ensure uniform tobacco quality. Additionally, the citrus extracts were diluted 30 times with water, and 30 mL of diluent was sprayed evenly on the surface of 200 g of CTLs. Then, the CTLs were evenly stacked in a constant temperature and humidity incubator at 35°C and 75% relative humidity for a fermentation period of 30 days. Accordingly, the CTL samples fermented with S1, S2, or S3 were designated as T1, T2, or T3, respectively. Besides, a blank control group without citrus extract was also included, with the CTLs named as T0.

### 2.4 Analysis of main chemical components in CTLs

The contents of protein, total nitrogen (TN), and alkaloids in CTLs were determined by a continuous flow analytical system according to the Tobacco Industry Standard. Amino acids and non-volatile organic acids (nVOCs) in CTLs were analyzed by High Performance Liquid Chromatography (HPLC), while aroma components were measured by Gas Chromatography-Mass Spectrometry (CG-MS). Details of the aforementioned analytical methods can be found in our previous studies (Hu et al., 2023a; Hu et al., 2023c). Each experiment was performed in triplicate.

### 2.5 Characterization of microbiota

CTL samples were collected after 30 days of fermentation for 16S rRNA and ITS high-throughput sequencing. Total DNA was extracted from the CTLs using PowerSoil^®^ DNA Isolation Kit (MO BIO Laboratories, Carlsbad, CA, United States) according to manufacturer’s instructions. Universal primer pairs 341F/805R were used to amplify the V3-V4 region of the 16S rRNA gene, and ITS-1F and ITS-1R primers were utilized to amplify the ITS1 region of fungi. Additional procedures were performed as outlined in our previous studies (Hu et al., 2023a).

### 2.6 Sensory evaluation of CTL samples

Five experts in cigar evaluation, possessing extensive experience in assessing cigar quality, were invited to participate in a sensory evaluation and analysis. The sensory quality assessment of the CTLs was conducted in strict accordance with the Chinese tobacco standard method. The fermented CTL samples were shaped into cigar sticks with a length of 90 mm and a circumference of 47 mm, and subsequently aged for 10 days in an aging cabinet (Bulldog VC528, China) maintained at a temperature of 20°C and a relative humidity of 60% for further sensory evaluation.

**FIGURE 1:**
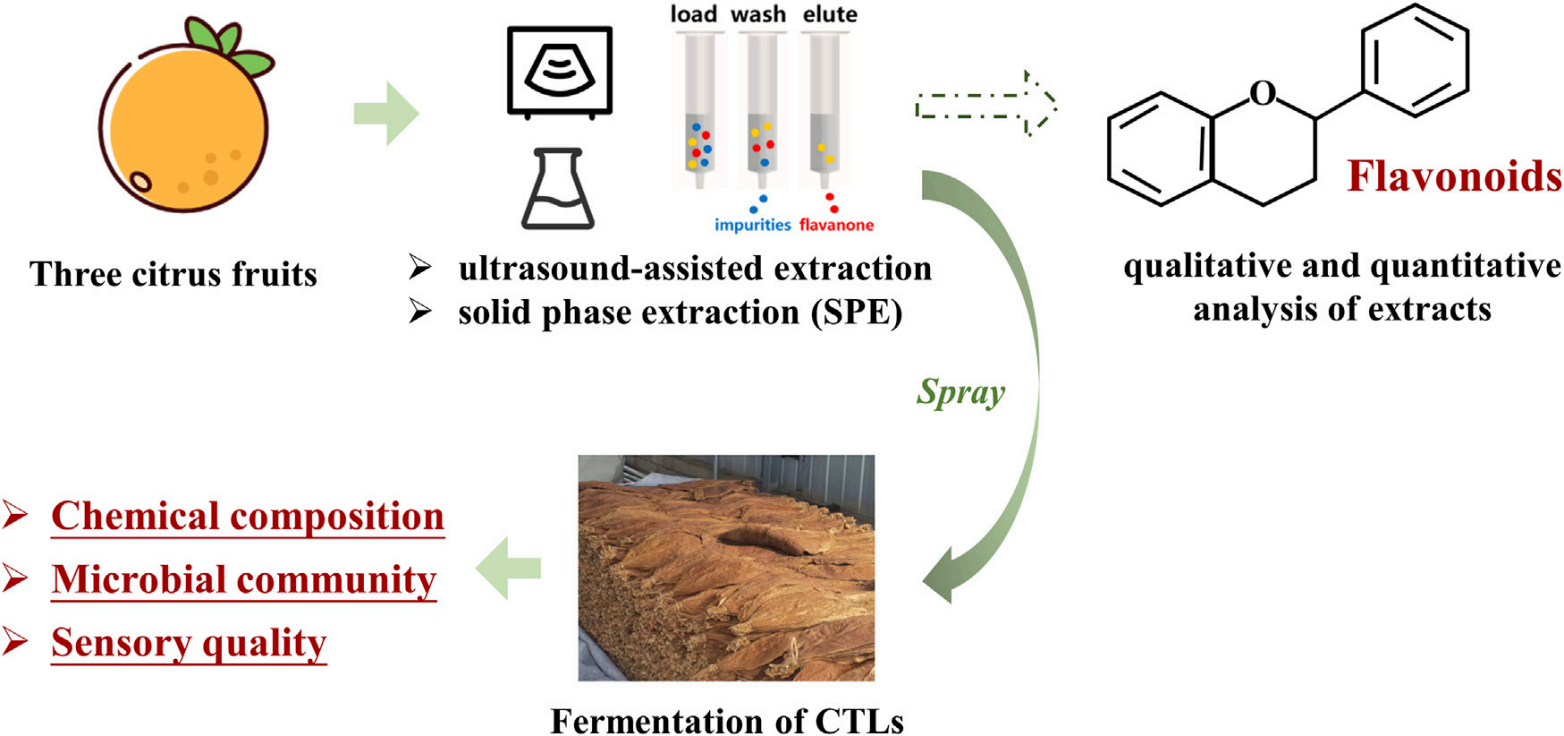
The procedure of extraction and fermentation experiments.

The experimental procedure employed in this study is summarized in Figure 1.

## 3 Results and discussion

### 3.1 Identification of main constituents in citrus extracts

**FIGURE 2:**
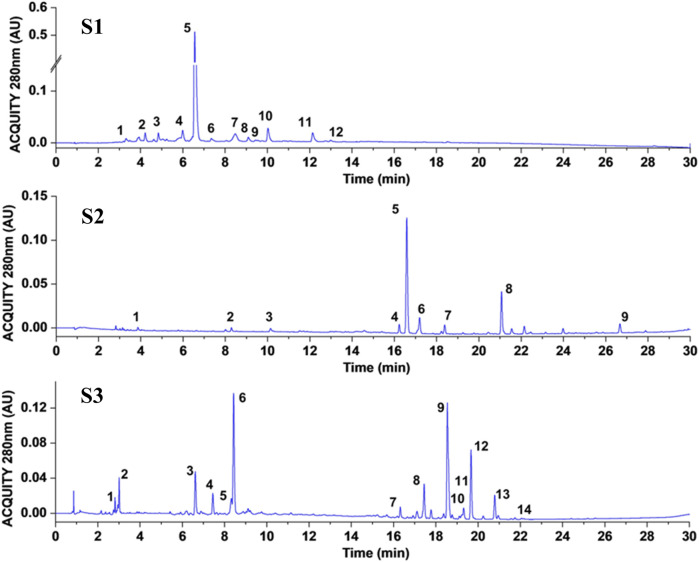
UPLC–ESI–MS/MS chromatograms of three citrus extracts.

The main constituents of S1 were identified and quantitatively analyzed by UPLC-ESI-MS/MS, with the resulting liquid chromatogram presented in Figure 2. Based on the retention time (RT) and MS data with those of reference standards, a total of ten polyphenols and two terpenoids were identified, as detailed in Table 2. The RT, molecular formula, quasi-molecular ions, and characteristic fragment ions are outlined in Table 2. The investigated compounds generally exhibited high-accuracy protonated/deprotonated molecular ions [M + H]^+^/[M−H]^−^ for analysis. Jiang et al. (2023) reported that flavonoids and monoterpene glucosides displayed high intensity in both positive and negative ionization modes. In this study, nine flavonoids were detected, including vicenin-2, luteolin-C-neohesperidoside, apigenin-8-C-neohesperidoside, 3′,5′-di-C-glucopyranosylphloretin, diosmin, acacetin-8-C-neohesperidoside, acacetin-6-C-neohesperidoside, acacetin-7-O-neohesperidoside, and poncirin, as listed in Table 2. Besides, the two identified terpenoids were limonin and ichangin. The aforementioned compounds in S1 were quantified, with the content of 3′,5′-di-C-glucopyranosylphloretin measured at 177.07 mg 100 g^-1^ dry weight (DW), accounting for 77.80% of the total quantified compounds. Consequently, both in terms of type and content, flavonoids were determined to be the predominant components of S1.

**TABLE 2:** Identification of chemical components and their contents in three citrus extracts by UPLC-ESI-MS/MS.

No.	RT (min)	Molecular formula	Positive ions (m/z)	Fragment ions (m/z)	Negative ions (m/z)	Fragment ions (m/z)	Identification	Content (mg 100 g^-1^ DW)
S1								
1	3.3178	C_21_H_32_O_10_	445.2187	467.1893, 265.1445, 247.1314	443.1901	nd	Dihydrophaseic acid glycosyl ester	1.97 ± 0.14
2	4.2213	C_27_H_30_O_15_	595.1665	617.1469, 577.1532, 457.1110	593.1524	473.1103, 353.0698	Vicenin-2	4.97 ± 0.31
3	4.8459	C_27_H_30_O_15_	595.1665	449.1093, 431.0971, 329.0669	593.1507	473.1014, 429.0885	Luteolin-C-neohesperidoside	5.56 ± 0.39
4	5.9939	C_27_H_30_O_14_	579.1705	601.1522, 561.2428, 433.1092	577.1556	413.0849, 293.0426	Apigenin-8-C-neohesperidoside	6.91 ± 0.41
5	6.5680	C_27_H_34_O_15_	599.1971	637.1476, 621.1797, 581.1865, 563.1739	597.1828	579.1685, 477.1409, 459.1304, 417.1201	3′,5′-di-C-glucopyranosylphloretin	177.07 ± 8.50
6	7.3563	C_28_H_32_O_15_	609.1814	631.1648, 463.1245, 301.1400	607.1678	nd	Diosmin	1.86 ± 0.05
7	8.4774	C_28_H_32_O_14_	593.1863	615.1710, 447.1297, 429.1195	591.1705	427.1095, 307.0609, 119.0348	Acacetin-8-C-neohesperidoside	10.14 ± 0.67
8	9.1024	C_28_H_32_O_14_	593.1863	615.1710, 447.1297, 413.2165, 285.0749	591.1718	471.1347, 427.1095, 307.0609, 119.0348	Acacetin-6-C-neohesperidoside	2.56 ± 0.11
9	10.0328	C_28_H_34_O_14_	595.2015	617.1825, 475.3274	593.1891	639.1917	Poncirin	8.39 ± 0.66
10	10.7518	C_26_H_30_O_8_	471.2007	493.1815, 409.1956	469.1893	515.1918	Limonin	0.24 ± 0.01
11	12.1480	C_28_H_32_O_14_	593.1863	615.1710	591.1705	637.1790	Acacetin-7-O-neohesperidoside	7.31 ± 0.56
12	12.9932	C_26_H_32_O_9_	489.2131	511.1935	487.1975	nd	Ichangin	0.69 ± 0.05
S2								
1	3.8668	C_27_H_30_O_15_	595.1604	577.1506, 559.1432, 379.1026, 295.0585	593.1552	575.1548, 503.1236, 473.1113, 297.0821	Vicenin-2	0.49 ± 0.02
2	8.2927	C_27_H_30_O_14_	579.1640	433.1226, 271.0569	577.1625	413.1364, 269.0465	Rhoifolin	0.57 ± 0.02
3	10.1582	C_15_H_10_O_5_	271.0575	153.0182, 119.0203, 91.0126	269.0476	227.0379, 151.0231, 107.0411	Apigenin	0.53 ± 0.04
4	16.2302	C_27_H_32_O_14_	581.1848	527.1632, 383.1061, 273.0724, 195.0269	579.1782	459.1205, 313.1028, 151.0035, 119.0856	Narirutin	1.91 ± 0.12
5	16.5880	C_27_H_32_O_14_	581.1808	435.1426, 419.1300, 315.0869, 273.0725	579.1755	459.1173, 373.0950, 271.0605, 193.0628	Naringin	37.12 ± 1.56
6	17.1923	C_15_H_12_O_5_	273.0734	153.0171, 147.0433, 123.0510, 119.0486	271.0606	177.0187, 151.0029, 119.0501, 107.0147	Naringenin	3.31 ± 0.21
7	18.3808	C_28_H_32_O_16_	625.1724	607.1573, 487.1167, 439.1033, 317.0643	623.1686	533.1122, 503.1242, 383.0800, 312.0662	Diosmetin-6,8-di-C-glucoside	1.91 ± 0.15
8	21.0718	C_28_H_32_O_15_	609.1778	301.0677, 286.0449	607.1754	299.0580, 284.0338	Neodiosmin	8.04 ± 0.39
9	26.6731	C_27_H_32_O_15_	597.1753	579.1625, 451.1230, 235.0622, 147.0524	595.1723	475.1138, 431.1033, 287.0576, 166.9979	Eriocitrin	1.77 ± 0.07
S3								
1	2.8202	C_16_H_18_O_9_	355.1576	163.0379, 145.0578, 135.0458	353.1024	191.1868, 93.1285, 85.1035	Chlorogenic acid	1.88 ± 0.24
2	3.0150	C_10_H_10_O_4_	195.1578	177.1575,145.3658	193.0505	160.8440, 133.0289, 118.9376	Ferulic acid	5.32 ± 0.69
3	6.6144	C_27_H_30_O_15_	595.1487	287.5831, 309.5481	593.2026	285.0158, 309.0551, 338.03967, 351.0848	Kaempferol rutinoside	14.32 ± 0.86
4	7.4411	C_27_H_32_O_14_	581.1880	383.1131, 273.0763	579.1792	459.1174, 271.0624	Naringin	6.45 ± 0.39
5	8.3179	C_28_H_34_O_15_	611.1888	303.1578	609.1866	301.1072, 286.0481	Hesperidin	3.26 ± 0.16
6	8.4279	C_28_H_34_O_15_	611.1980	413.1249, 303.0873	609.1876	301.1154, 286.3236	Neohesperidin	38.67 ± 5.41
7	16.3182	C_20_H_20_O_7_	373.1289	357.0979, 343.0816, 329.0872, 153.0187	371.1287	355.0548, 329.1575	Sinensetin	0.93 ± 0.13
8	17.4459	C_21_H_24_O_8_	405.1556	241.0716, 226.0485, 211.0248, 183.0296	403.1548	241.0186, 226.0587	Citromitin	9.85 ± 0.59
9	18.5421	C_21_H_22_O_8_	403.1392	388.1147, 373.0914, 355.0815, 327.0852	401.0248	386.2486, 371.5786, 325.454	Nobiletin	41.18 ± 2.88
10	18.7703	C_19_H_18_O_8_	375.1081	360.0849, 345.0609, 330.0381, 327.0508	373.7557	358.1575, 343.1574	Ternatin	0.86 ± 0.04
11	19.2460	C_22_H_24_O_9_	433.1508	418.1268, 403.1024, 388.0804, 345.0617	431.1832	415.1482, 401.1548, 386.0548	3-methoxynobiletin	2.79 ± 0.33
12	19.6596	C_20_H_20_O_7_	373.1280	358.1040, 343.0804, 328.0575, 300.0628	371.1576	341.15743, 326.4578	Tangeretin	29.01 ± 3.48
13	20.7832	C_20_H_20_O_8_	389.1232	374.1004, 359.0763, 341.0658, 197.0086	387.1258	357.0546, 338.1547	5-demethylnobiletin	5.82 ± 0.70
14	22.0453	C_19_H_18_O_7_	359.1135	344.0898, 329.0655, 311.0553, 197.0084	357.0547	342.1584, 327.5823,309.1552	5-hydroxy-7,8,3′,4′-tetramethoxyflavone	0.25 ± 0.03

In a similar manner, we performed both qualitative and quantitative analyses for samples S2 and S3. As illustrated in Figure 2 and Table 2, a total of nine compounds were identified in S2. Seven flavonoids were definitively identified by comparison with available reference standards, while the remaining two were characterized based on their accurate molecular weights and mass spectrometric properties. Based on their structural characteristics, four of the flavonoids were classified as flavanones (namely, narirutin, naringin, naringenin, and eriocitrin), whereas the other five were categorized as flavonoids (specifically, vicenin-2, rhoifolin, apigenin, diosmetin-6,8-di-c-glucoside, and neodiossmin). As detailed in Table 2, naringin and neodiosmin were found to be the most abundant compounds, with contents of 37.12 and 8.04 mg 100 g^-1^ DW, respectively, which correspond to 66.72% and 14.45% of the total compounds detected.

In addition, a total of fourteen polyphenolic compounds, including twelve flavonoids and two phenolic acids, were identified in S3, as detailed in Table 2. The two phenolic acids present in S3 were ascertained as chlorogenic acid and ferulic acid. Based on their basic fundamental structures and functional substituents, the twelve flavonoids were classified as follows: one flavonol (kaempferol rutinoside), three flavanones (naringin, hesperidin, and neohesperidin), seven flavones (sinensetin, nobiletin, ternatin, 3-methoxynobiletin, tangeretin, 5-demethylnobiletin, and 5-hydroxy-7,8,3′,4′-tetramethoxyflavone), and one flavonone (citromitin). Notably, nobiletin and neohesperidin exhibited the highest content of 41.18 and 38.6 mg 100 g^-1^ DW. Furthermore, the flavonoids showed a relatively high content, especially the polymethoxylated flavonoids, which contain more than four methoxy groups (including sinensetin, nobiletin, tangeretin, and 5-demethylnobiletin).

In summary, flavonoids represent the primary component of the three citrus extracts analyzed. As the main constituent, flavonoids have been previously reported to exhibit antioxidant activity (Sang et al., 2021). Furthermore, flavonoids are significant components in tobacco leaves, serving as aroma precursors and playing a crucial role in regulating the quality of tobacco leaves (Qi et al., 2023). Consequently, it can be posited that the quality of CTLs may be improved through the incorporation of citrus flavonoids during the fermentation of CTLs.

### 3.2 Alkaloids, TN, amino acid, and protein of CTLs

**FIGURE 3:**
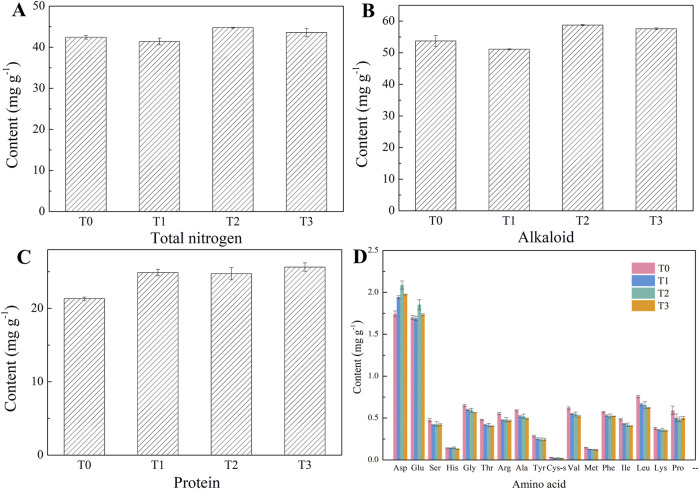
Contents of total nitrogen **(A)**, alkaloid **(B)**, protein **(C)**, and amino acid **(D)** in different cigar tobacco leaves.

As shown in Figure 3, the content of TN in CTLs ranged from 41.39 to 44.75 mg g^-1^. Notably, the TN content in the T2 sample was higher than that in T0, reflecting an increase of 5.51%. In contrast, the TN content in T1 and T3 did not exhibit a statistically significant difference compared to T0. Regarding alkaloid content, a decrease of 4.95% was observed in T1 relative to T0, while T2 and T3 demonstrated increase in 9.34% and 7.34%, respectively. Furthermore, the incorporation of citrus extracts resulted in an increase in protein content across the three CTL samples, with increase of 6.71%, 15.99%, and 20.19%, respectively.

As illustrated in Figure 3D, the total content of amino acids in CTLs ranged from 9.56 to 10.20 mg g^-1^. The addition of citrus extracts resulted in a reduction of the total content of amino acids in T1, T2, and T3 by 5.55%, 2.85%, and 7.01%, respectively. Amino acids are recognized as significant nitro-containing compounds in tobacco leaves. In addition to participating in the Maillard reaction with reduced sugars to produce flavor components such as pyrazine and pyrrole, some amino acids can also decompose themselves to produce flavor substances (Charnock et al., 2022). Therefore, amino acids showed an important impact on the quality of CTLs (Huang et al., 2023). It is widely accepted that an excess of free amino acids in tobacco leaves can lead to smoke irritation and an unpleasant aftertaste (Chen et al., 2024). Therefore, the decrease of amino acids in this study was favorable for mitigating the irritation of CTLs. Among the seventeen amino acids detected, aspartic acid (Asp) and glutamic acid (Glu) had the highest content, collectively accounting for approximately 30%–40% of the total content of amino acids. Furthermore, the addition of the three citrus extracts resulted in an increase in the Asp content in CTLs, while the levels of other amino acids declined with the addition of citrus extracts.

### 3.3 Organic acid of CTLs

Organic acids and their derivatives play a significant role in the flavor profile of tobacco. These acidic compounds are generated during the combustion and smoking processes, contributing to the pH balance of smoke, improving the mellowness of taste, and reducing irritation (Yang et al., 2023). Analysis of nVOCs of CTLs fermented with citrus extracts identified a total of 14 compounds, as detailed in Table 3. The total content of nVOCs ranged from 9,819.94 to 12,142.18 μg g^-1^. Notably, the incorporation of citrus extract appeared to have a minimal impact on the overall content of nVOCs.

**TABLE 3:** Contents of non-volatile organic acids in CTLs.

Content (μg g^-1^)	T0	T1	T2	T3
Lactic acid	9.15 ± 2.66	7.84 ± 0.29	8.83 ± 0.09	8.33 ± 0.56
Oxalic acid	2,204.37 ± 157.38	2008.23 ± 49.83	1995.87 ± 33.52	1866.76 ± 13.22
Malonic acid	226.29 ± 11.78	228.02 ± 4.48	214.17 ± 2.91	223.16 ± 1.25
Levulinic acid	12.36 ± 1.02	14.89 ± 0.59	19.07 ± 0.46	13.27 ± 0.13
Succinic acid	200.71 ± 8.22	201.20 ± 5.64	159.99 ± 3.60	155.45 ± 3.94
Malic acid	1,669.07 ± 202.79	1,068.45 ± 340.98	1775.79 ± 341.29	1,354.29 ± 172.46
Citric acid	5,515.04 ± 243.88	5,997.01 ± 65.07	6,240.54 ± 413.22	8,179.40 ± 327.23
Vanillic acid	58.80 ± 2.54	56.13 ± 4.46	61.02 ± 5.99	57.38 ± 11.49
Myristic acid	18.85 ± 2.60	17.22 ± 4.04	17.93 ± 4.87	25.60 ± 8.78
Palmitic acid	110.29 ± 1.83	90.13 ± 34.33	82.25 ± 4.94	82.54 ± 4.15
Linoleic acid	60.07 ± 2.03	34.44 ± 1.61	41.39 ± 1.76	47.48 ± 7.63
Oleic acid	117.56 ± 2.53	71.27 ± 2.64	86.61 ± 4.74	97.28 ± 10.15
Stearic acid	22.93 ± 1.37	18.48 ± 1.32	18.63 ± 1.48	22.30 ± 5.85
Eicosanoic acid	4.68 ± 0.62	6.62 ± 1.21	9.34 ± 4.48	8.95 ± 3.70
Saturated fatty acid	156.76 ± 2.60	132.44 ± 30.65	128.15 ± 4.69	139.37 ± 19.29
Unsaturated fatty acid	177.64 ± 4.56	105.72 ± 4.19	127.99 ± 6.44	144.77 ± 17.33
Total	10,230.19 ± 337.25	9,819.94 ± 348.82	10,731.41 ± 504.61	12,142.18 ± 447.14

Previous studies have proved that malic acid, citric acid, and oxalic acid were the main components of organic acids in tobacco leaves (Lu et al., 2021; Zong et al., 2023). In this study, the content of oxalic acid in CTLs ranged from 1866.76 to 2,204.37 μg g^-1^, malic acid content ranged from 1,068.45 to 1775.79 μg g^-1^, and citric acid content ranged from 5,515.04 to 8,179.40 μg g^-1^. Collectively, these three acids constituted over 90% of the total nVOCs.

It was found that six NOCs, including lactic acid, oxalic acid, succinic acid, malic acid, linoleic acid, and oleic acid, showed a decreasing trend with the addition of citrus extracts. Conversely, there were two nNOCs with a positive correlation, *i.e.*, citric acid and eicosanoic acid. These acids influenced the sensory quality of CTLs in various ways. Excessive oxalic acid was known to impart an astringent taste and cause irritation to smoke, so the decrease of oxalic acid content in T1, T2, and T3 samples was conducive to reducing the irritation of CTLs. Besides, the content of citric acid in T1, T2, and T3 increased by 8.74%, 13.15%, and 48.31%, respectively. Although citric acid is a naturally occurring component in tobacco, it was often adopted as a tobacco additive. This is because citric acid can reduce the amount of nicotine entering the smoke, thus reducing the irritation of the smoke and improve the taste (Zong et al., 2023).

Notably, compared with T0, the content of unsaturated fatty acids in T1, T2, and T3 decreased by 15.51%, 18.25%, and 11.09%, respectively. The relatively elevated levels of unsaturated fatty acids in cigars would increase the irritation and roughness of smoke. Based on the aforementioned results, it can be inferred that the contents of various organic acids in citrus extract-treated CTLs underwent significant alterations during fermentation, which facilitated a reduction in irritation and an improvement in the mellowness of CTLs.

### 3.4 Aroma component of CTLs

**FIGURE 4:**
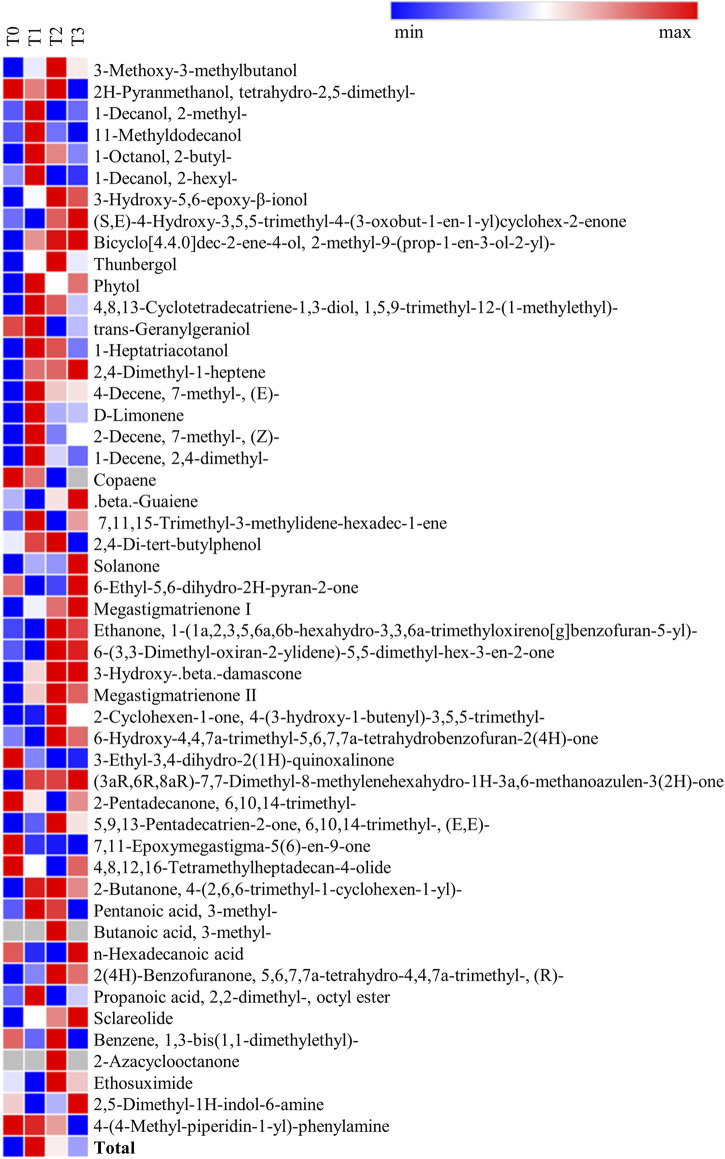
Heatmap cluster of aroma compounds in different CTL samples.

The flavor profile, including the composition and content of aroma components, is a significant indicator of CTLs quality. In our analysis, we identified a total of 51 flavor compounds, including 14 alcohols, eight alkenes, 16 ketones, 3 acids, 3 esters, and 6 other compounds (Figure 4). Results from GCMS showed a significant impact on the aroma composition due to the fermentation of CTLs with citrus extracts. Notably, the total content of all aroma components in T0 was obviously lower than that in T1, T2, and T3 samples, with observed increases of 38.15%, 20.45%, and 12.06%, respectively. It is essential to emphasize that fermentation with citrus extracts has positively influenced the content of aroma components, potentially enhancing the overall aroma of CTLs. Our previous research has also demonstrated improvements in the aroma profile of CTLs fermented with exogenous additives (Hu et al., 2023b).

Furthermore, the contents of alkenes, alcohols, and esters in the T1, T2, and T3 samples were significantly higher than those in the T0 sample. Specifically, the content of alcohols increased by 104.63%, 85.30%, and 47.40%, respectively. Conversely, the T0 sample exhibited higher levels of ketones and nitrogen-containing heterocyclic compounds than the other samples. In contrast, no significant differences were observed in the total content of acids among the four samples. In detail, the CTLs fermented with citrus extracts exhibited a relatively higher content of several compounds, including 2-butyl-1-octanol, 2-hexyl-1-decanol, 3-hydroxy-5,6-epoxy-β-ionol, phytol, 1-heptatriacotanol, 2,4-dimethyl-1-heptene, (E)-7-methyl-4-decene, *D*-limonene, (Z)-7-methyl-2-decene, 2,4-dimethyl-1-decene, 7,11,15-trimethyl-3-methylidene-hexadec-1-ene (neophytadiene), 4-(2,6,6-trimethyl-1-cyclohexen-1-yl)-2-butanone (β-dihydroionone), and (R)-5,6,7,7a-tetrahydro-4,4,7a-trimethyl-2(4H)-benzofuranone (dihydroactinidiolide). The increase in the contents of these substances was particularly significant to improve the flavor of CTLs, as they are recognized as important flavor components in cigars (Ren et al., 2023). For instance, *D*-limonene imparts fruity and refreshing aromas to CTLs, while neophytadiene, a neutral aroma substance, is present in high content in tobacco and can direct influence the taste and aroma of tobacco leaves (Yao et al., 2022). Additionally, 2-butyl-1-octanol, a C8 alcohol, is noted for its aromatic qualities. The compound of 3-hydroxy-5,6-epoxy-β-iono is an intermediate product resulting from the degradation of carotenoids. Additionally, although the ketone content decreased in CTLs fermented with citrus extracts, the content of β-dihydroionone increased; this compound is significant fragrance derived from the degradation of carotenoids and is characterized by floral, woody, and fruity aromas (Yang et al., 2024).

In conclusion, the addition of citrus extract in this study facilitated the accumulation of aroma substances in CTLs and increased the content of several characteristic aroma components, such as β-dihydroionone, which is of great significance for improving the aroma of CTLs.

### 3.5 Microbial community of CTLs

A deep analysis of bacterial and fungal communities presented in the four CTL samples is crucial to understand the influence of citrus extracts. The microbial diversities within these samples were investigated, revealing that the coverage for each sample exceeded 99.74% (Table 4). These high coverages indicates that the sequencing depth for each sample was sufficient to reflect the bacterial and fungal compositions.

**TABLE 4:** Richness and diversity of microbial communities in CTLs.

Sample	Shannon index		Simpson index		Ace index		Chao index		Coverage (%)	
	Bacteria	Fungi	Bacteria	Fungi	Bacteria	Fungi	Bacteria	Fungi	Bacteria	Fungi
T0	1.50	2.70	0.52	0.13	507.66	714.14	482.96	697.18	99.80	99.86
T1	1.39	2.56	0.56	0.14	506.80	714.74	479.04	694.73	99.74	99.84
T2	1.58	2.44	0.53	0.16	515.31	689.36	495.79	674.68	99.79	99.84
T3	1.18	2.70	0.66	0.12	526.11	726.28	505.75	699.54	99.81	99.88

We further evaluated the bacterial and fungal diversity of the four CTL samples based on Ace, Chao, Shannon, and Simpson indexes. As shown in Table 4, the T3 sample exhibited the highest Ace and Chao indexes for both bacteria and fungi among the four samples, suggesting an increase in the richness of bacterial and fungal communities in the CTL samples following the addition of *Citrus Yuzu L.* extract. Furthermore, the Shannon index revealed that the bacterial diversity of T1 and T3 samples was lower than that of T0, whereas the bacterial diversity of T2 was found to be higher. Additionally, it was noted that the fungal diversity of CTLs fermented with citrus extracts experienced a decline.

**FIGURE 5:**
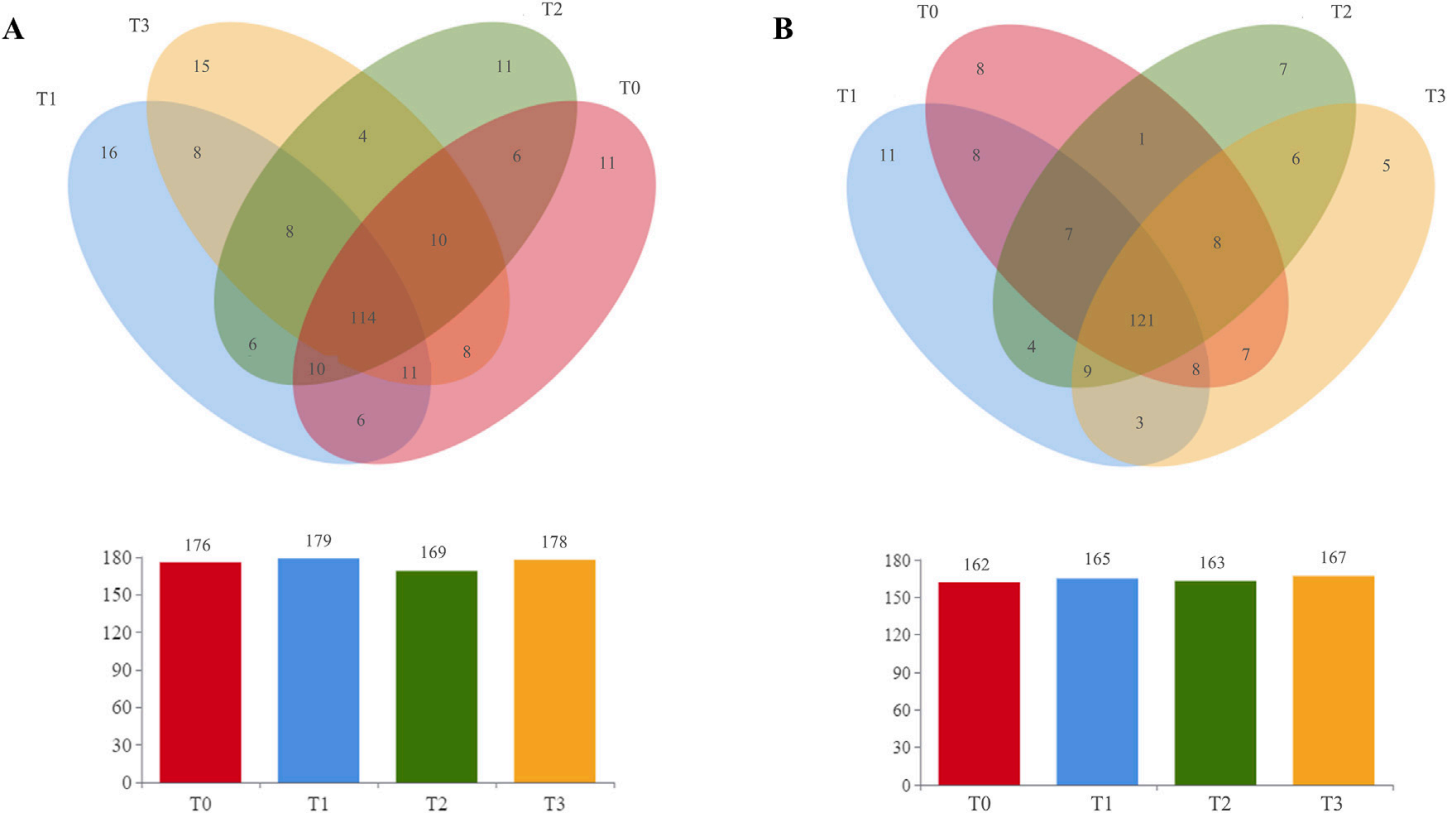
The number of **(A)** bacterial OTUs and **(B)** fungal OTUs in different groups.

The operational taxonomic units (OTUs) of four CTL samples were compared and analyzed. As shown in Figure 5, a total of 244 bacterial OTUs and 213 fungal OTUs were identified in the CTLs. Among these, 114 bacterial OTUs and 121 fungal OTUs were found to be common across the samples, accounting for 46.72% and 56.81% of the total bacterial and fungal OTUs, respectively. This finding suggests that these shared bacterial and fungal groups play an important role in the fermentation of CTLs. Furthermore, the analysis revealed that the four CTL samples T0-T3 contained 11, 16, 11, and 15 unique bacterial OTUs, as well as 8, 11, 5, and 7 unique fungal OTUs, respectively. These results indicated that the addition of citrus extracts accelerated the succession of microbial communities in CTLs.

**FIGURE 6:**
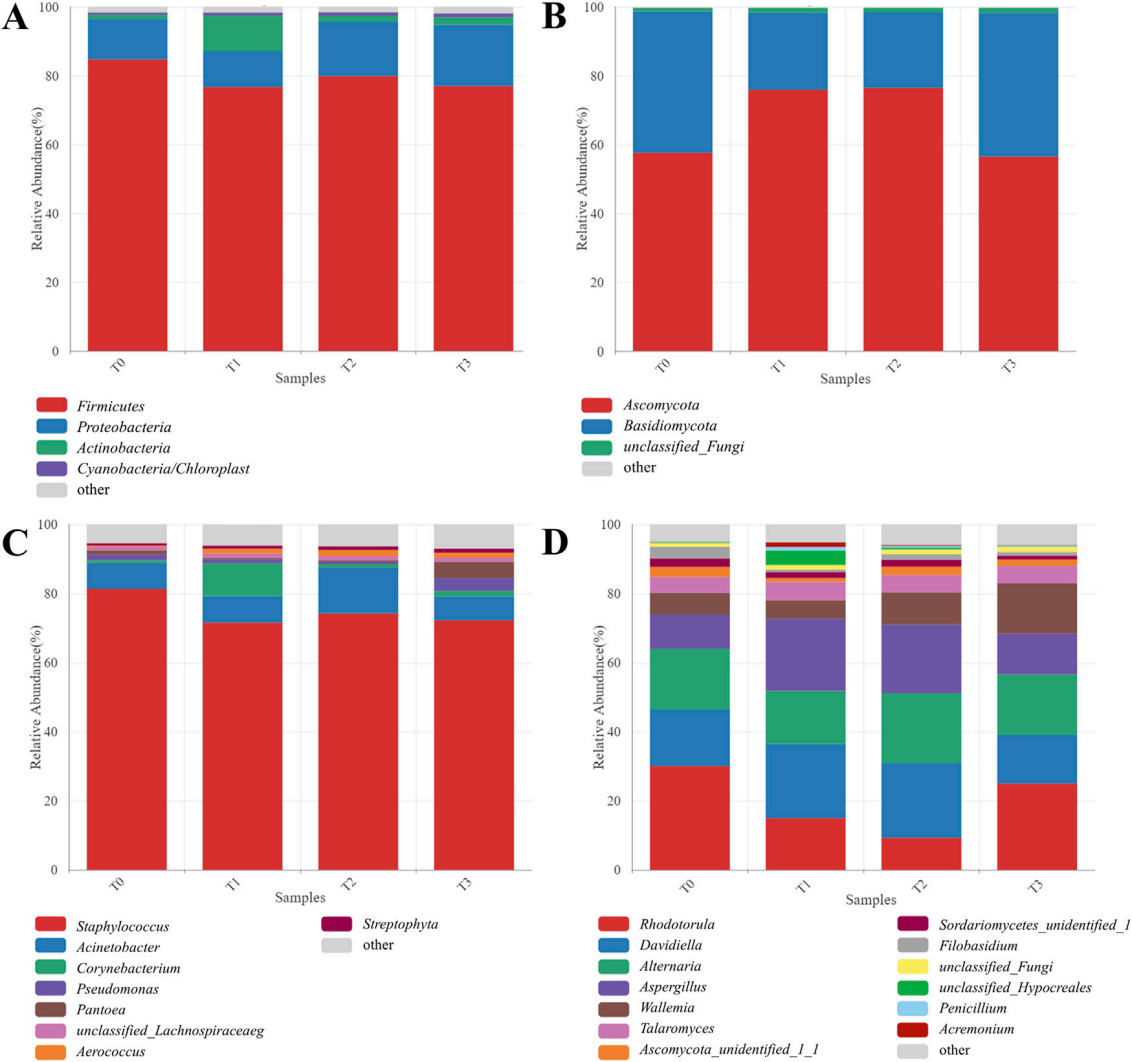
Microbial community composition of CTL samples. The relative abundance of bacterial taxon at **(A)** phylum and **(C)** genus level, respectively. The relative abundance of fungal taxon at **(B)** phylum and **(D)** genus level, respectively.

The microbial composition of the four CTL samples is shown in Figure 6. Regarding the bacterial community at the phylum level, Firmicutes emerged as the most abundant taxon, constituting over 70% of the bacterial community (Figure 6A), which is consistent with previous results (Ren et al., 2023; Zhang et al., 2019). Additionally, Proteobacteria were identified as another dominant bacterial group in tobacco leaves. Both Firmicutes and Proteobacteria played an important role in the carbon degradation during tobacco fermentation, such as the degradation of starch, cellulose, and pectin to maltose, fructose, and glucose (Costa et al., 2020). The genus *Staphylococcus* was the most prevalent across all samples, with relative abundances of 81.39% in T0, 71.65% in T1, 74.29% in T2, and 72.31% in T3. This trend indicates a decrease in the abundance of *Staphylococcus* in CTLs with the addition of citrus extracts. Although *Staphylococcus* has been widely reported as the predominant bacterial genus in tobacco leaves, the mechanisms by which it influences the quality of tobacco leaves remain underexplored in the literature (Zheng et al., 2022a). Furthermore, *Acinetobacter* was dominant in T2 (13.18%), followed by T1 (7.65%), T0 (7.52%), and T3 (6.84%). Of particular interest, *Corynebacterium* was identified at a significant level of 9.54% in T1, which was significantly different from the other three samples. *Corynebacterium* has been reported to produce enzymes capable of generating various acids through the breakdown of different sugars (Wieschalka et al., 2013). It has been proved that *Corynebacterium* can be inoculated with tobacco extract to produce flavor-enhancing extract, which can be used to improve the baked sweetness of tobacco leaves (Xu et al., 2021). Therefore, the increased abundance of *Corynebacterium* was favorite for the sensory quality of CTLs. Moreover, the abundance of *Pseudomonas* (3.81%) and *Pantoea* (4.61%) were found in T3. *Pseudomonas* was reported to show superior capacity to degrade nicotine, and a reduction in nicotine content may lead to decreased irritation of CTLs (Raman et al., 2014).

Ascomycota and Basidiomycota were the predominant phyla in fungal community, collectively accounting for over 95% of the total abundance (Figure 6B). *Rhodotorula*, *Davidiella*, *Alternaria*, *Aspergillus*, and *Wallemia* were the dominant genera in all four samples (Figure 6D). Significant differences were observed in the fungal communities among the four samples. The relative abundance of *Rhodotorula* was highest in T0 (30.09%), followed by T3 (25.02%), T1 (15.00%), and T2 (9.34%), whereas *Aspergillus* exhibited its highest abundance in T1 (20.93%), followed by T2 (19.89%), T3 (11.80%), and T0 (9.82%). Many fungal strains of *Rhodotorula* have been reported to have a positive correlation with the production of carotenoids, oils, and esters (Garay et al., 2017). Additionally, certain *Aspergillus* species isolated from tobacco leaves possessed polysaccharide-degrading enzyme activity and the potential to improve the quality of tobacco leaves (Zhang et al., 2024). Notably, T2 exhibited the highest abundance of *Alternaria* (20.22%) and *Davidiella* (21.65%), while T3 displayed the highest abundance of *Wallemia* (14.56%). *Alternaria* and *Wallemia* existed commonly in tobacco, since *Alternaria* was found displayed a positive correlation with sugar contents (Si et al., 2023), while *Wallemia* was positively associated with various volatile compounds, including isoamyl alcohol, 1-hexanol, 2-octanol, acetic acid, guaiacol, and tetramethylpyrazine (Du et al., 2022).

In conclusion, alterations in functional microorganisms, such as C*orynebacterium* and *Pseudomonas*, alongside the modifications in microbial communities in CTLs after fermentation with citrus extracts, contributed to a reduction of irritation and an enhancement of aroma substances in CTLs.

### 3.6 Sensory evaluation of CTLs

Aside from analyzing chemical compositions and microbial communities, it is essential to understand the perceived attributes and acceptance by professional evaluators in order to assess the influence of citrus extracts on the characteristics of cigar tobacco. Thus, a macroscopic analysis was conducted to evaluate the impact of citrus extracts on the sensory quality of CTLs. Fermented CTLs were prepared for the assessment of aroma, smoke characteristics, aftertaste, and overall acceptability.

Based on a comprehensive analysis (Table 5), CTLs fermented with water (T0) showed weak aroma characterized by obvious impurity and irritation of smoke. Therefore, it is significant to optimize the fermentation process to reduce the irritation and increase the aroma richness of CTLs. Notably, decreased irritation was all observed in samples T1, T2, and T3. Among these, T1 demonstrated significant improvements in aroma and flavor attributes, as well as smoke characteristics, with a marked decrease in irritation and an enhancement in sweetness, maturity, and aroma richness. T2 exhibited increased mellowness, sweetness, and maturity. T3 showed decreased smoke concentration and improved matureness. Besides, the fruit aroma was also enhanced in all three samples. The improvement of aroma richness in the fermented CTLs may be attributed to an increase in specific aroma components, such as *D*-limonene, β-dihydroionone, and 3-methyl-pentanoic acid, which impart wood, flower, and fresh lemon aromas (Figure 4). Furthermore, the increase in dihydroactinidiolide, along with a decrease in unsaturated fatty acids and oxalic acid, contributed positively to the reduction of irritation in CTLs.

**TABLE 5:** Effects of different citrus extracts on sensory quality of CTLs.

Sample	Overall evaluation
T0	Obvious irritation was found, the fragrance was not abundant, the smoke concentration and strength were large, and the aftertaste was uncomfortable
T1	Aroma and smoke characteristics were improved: reduced irritation, as well as improved sweetness, matureness, and aroma richness were observed
T2	Decreased irritation as well as increased mellowness, sweetness, and matureness were found
T3	Smoke concentration and irritation were reduced, while matureness and aroma richness were improved

In summary, the results obtained from instrumental analysis regarding irritation and aroma were consistent with those from the sensory evaluation. These findings suggest that the fermentation of CTLs with citrus extracts diminished the pungent odor while enhancing the aroma richness of CTLs.

## 4 Conclusion

In this study, three types of active ingredients derived from citrus were obtained through a multistage extraction and separation process utilizing citrus plants as raw materials. These ingredients were employed as exogenous additives in the fermentation of CTLs. The results showed that flavonoids constituted the primary components of the three citrus extracts. The addition of these extracts resulted in a reduction of amino acids, oxalic acid, and unsaturated fatty acids, while simultaneously increasing the content of citric acid. Additionally, the levels of aromatic compounds, particularly notable representative aroma components such as *D*-limonene and β-dihydroionone, were increased. Moreover, the addition of citrus extracts promoted the succession of microbial communities and the enrichment of microorganisms such as C*orynebacterium* and *Pseudomonas* in CTLs. From the perspective of sensory quality, the addition of citrus extracts significantly reduced irritation and improved the aroma quantity and richness of CTLs.

## Data Availability

The datasets presented in this study can be found in online repositories. The names of the repository/repositories and accession number(s) can be found below: https://www.ncbi.nlm.nih.gov/, PRJNA1138935 https://www.ncbi.nlm.nih.gov/, PRJNA1138927.
